# Potentially Functional Apple Snacks Infused in the *Hibiscus sabdariffa* Extract Obtained by Convective and Infrared Drying: Kinetics of Drying and Phytochemical Analysis

**DOI:** 10.1002/fsn3.70060

**Published:** 2025-03-04

**Authors:** Lavinia Stan (Boldea), Gabriel – Dănuț Mocanu, Mihaela Turturică, Doina Georgeta Andronoiu, Gabriela Râpeanu, Nicoleta Stănciuc

**Affiliations:** ^1^ Faculty of Food Science and Engineering ”Dunărea de Jos” University of Galați Galați Romania

**Keywords:** apple, browning, drying, hibiscus, mathematical models, value‐added

## Abstract

Apple snacks were obtained by using convective air (CD) and infrared drying (IR). The apple slices were immersed in *Hibiscus sabdariffa* aqueous extract for 30 min, followed by drying at temperatures varying between 50°C and 70°C. The kinetics of drying data were analyzed based on higher values of *R* ^*2*^ and lower *SSR* values and fitted to the Page model. The effective moisture diffusivity was higher for infused samples. The color of the infused sample increases, whereas the other colorimetric indices denote the appearance of enzymatic and non‐enzymatic browning. The browning index indicated a combined inhibitory effect of *Hibiscus* extract and temperature, up to 70°C, on oxidative enzymes. Regardless of the drying method, the infused samples showed significantly lower firmness and increased crispness. The rehydration and shrinkage ratio showed the highest value after 5 min of drying at 70°C, with higher values for IR. Color was highly appreciated for the infused variants, with the highest overall acceptability scores for the variants obtained by drying at 70°C. The antioxidant activity of all samples showed no statistically significant differences (*p* < 0.05), with the highest value of 3.19 ± 0.03 mMol/g DW for the infused samples dried by CD at 50°C. Cafestol was the main compound found in all samples; moreover, the infused samples showed higher values for bioactive compounds due to the extract's contribution to the polyphenolic profile of the samples. The potentially functional properties of the infused apple snacks were appreciated based on the phytochemical profile, antioxidant activity, color, and textural properties.

## Graphical abstract

Apple snacks were obtained by using convective air (CD) and infrared drying (IR). The apple slices were immersed in the *Hibiscus sabdariffa* aqueous extract for 30 min, followed by drying at temperatures varying between 50°C and 70°C. The potentially functional properties of the infused apple snacks were appreciated based on the phytochemical profile, antioxidant activity, color, and textural properties.

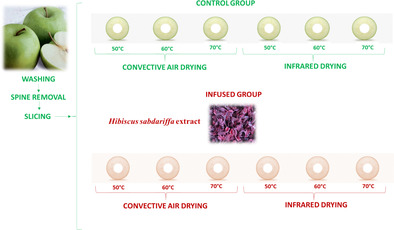

## 1 Introduction

A healthy and sustainable diet makes an important contribution to human health, especially due to the awareness of diet‐associated effects, leading to a greater interest in natural products, without additive additions, with beneficial effects (Zhu et al. 2022). Consequently, taking into account consumer preferences, fruit and vegetable snacks, as part of essential human daily diet, have an ever‐greater impact on the food industry and on the market. FAO (2021) recommendation for daily intake of fruits and vegetables is 200 g and can be considered vectors for valuable nutrients and bioactive compounds. Fruits are a source of dietary fiber, carbohydrates, healthy fats, proteins, as well as vitamins, minerals, and organic acids, providing, together with vegetables, the base of the food pyramid (Karwacka et al. 2022). Therefore, as suggested by Day et al. (2009), natural foods can be considered the simplest and easiest source of nutrients and important bioactives for human health, given the fact that the demand for functional foods with beneficial health effects is constantly increasing.

Although the consumption of fresh fruits remains an essential prerequisite for healthy nutrition, nowadays, snacking gains a significant role in the contemporary diet (de Gooijer et al. 2023). Unfortunately, in recent decades, snack consumption significantly contributes to a higher total energy intake, especially due to the presence of high quantities of fats and sugar (Murakami and Livingstone 2015).

Apple ( *Malus domestica* ) is a popular and ancient fruit of the *Myrtaceae* family. From a compositional point of view, the apple was extensively studied due to its beneficial health‐related nutritional and phytochemical content, such as vitamins, sugars, polysaccharides, dietary fiber, organic acids, polyphenolic compounds (tannins), carotenoids, sterols, and mineral elements essential for human nutrition (Zhu et al. 2022). Due to its specific properties and the high content of bioactive compounds, the apple is considered an ideal fruit to deliver snacks in relation to the consumption profile of the latter, bringing beneficial impacts on blood pressure, inflammation, vascular function, hyperglycemia, and the potential to limit cholesterol increase (Arora et al. 2018; Bondonno et al. 2017).

However, when processing, due to the high enzymatic activity of polyphenol oxidase (PPO) and peroxidase (POD), the enzymatic browning highly affects the quality of apple products, especially due to the destruction of the spatial cellular regionalized structure of phenols by the specific action of enzymes, leading to dark brown substances (Xu et al. 2022), simultaneously with shelf‐life reduction due to quality degradation. In order to inhibit the enzymatic activity of PPO and POD, different postharvest treatments are currently applied, based on physical and chemical treatments, including low and high temperature, packaging technology, high pressure, ultraviolet irradiation, and the use of browning inhibitors (Wang et al. 2023). Currently, our research group demonstrated that natural polyphenols could inhibit about 50% of PPO activity (Stan (Boldea), Aprodu, et al. 2023) by using an infusion step of fresh‐cut apples in the aqueous extract of *Hibiscus sabdariffa* L. From our results, it can be seen that *Hibiscus sabdariffa* L. extract contains a wide range of polyphenols, such as gallic acid, caffeic acid, syringic acid, sinapic acid, hibiscus acid, azelaic acid, 3,4‐dihydroxybenzoic acid, chlorogenic acid, ferulic acid, ellagic acid, *p*‐coumaric acid, neochlorogenic acid, delphinidin‐3‐*O*‐sambubioside/hibiscin, delphinidin, pelargonidin, delphinidin‐3‐*O*‐β‐D‐glucoside, kaempferol, kaempferol‐3‐*O*‐sambubioside, kaempferol‐3‐*O*‐rutinoside, naringenin, myricetin, gallocatechin, kaempferol glucoside, and spicoside A [Stan (Boldea), Aprodu, et al. 2023].

Therefore, the main objective of our study was to obtain apple snacks with a reduced browning index by infusing the fresh‐cut apples in aqueous extract of *Hibiscus sabdariffa* L., followed by drying, using two methods, convective air drying (CD) and infrared drying (IR), respectively. Omolola et al. (2017) explained that drying significantly contributes to the extension of the shelf life by minimizing the water concentration in the product, thus impacting logistics costs. Hence, the effects of drying methods and different temperatures, such as drying at 50°C, 60°C, and 70°C on drying kinetics, polyphenol contents, and antioxidant activity were studied. Additionally, the study included selected physical characteristics in order to evaluate the acceptability of the products, such as rehydration ratio (RR) and shrinkage ratio (SR), surface area, texture characteristics, color parameters, and sensory analysis. The advanced polyphenolic content in terms of phenolic acids, flavonoids, and anthocyanins was analyzed by chromatographic methods. The obtained results are valuable in terms of developing innovative apple snacks with different natural colors and increased antioxidant activity.

## 2 Materials and Methods

### 2.1 Raw Material

Apples ( *Malus pumila* , cv. Granny Smith) were obtained from a local market in Galati, Romania, and stored at 4°C + 1°C prior to processing. The initial moisture content of apples was 81.16% ± 0.11%. *Hibiscus sabdariffa* dried calyx was purchased from a local herbal shop (Galați, Romania) in October 2023.

### 2.2 Reagents

The reagents used in this study were gallic acid, catechin, Trolox (6‐hydroxy‐2,5,7,8‐tetramethylchromane‐2‐carboxylic acid), Folin–Ciocalteu's reagent, 2,2‐diphenyl‐1‐picrylhydrazyl (DPPH), and ethanol, purchased from Sigma Aldrich Steinheim (Darmstadt, Germany). The reagents used in chromatographic analysis [acetic acid, acetone, acetonitrile, formic acid, methanol, ethanol, cafestol, procyanidin B1, procyanidin A1, gallic acid, caffeine, caffeic acid, syringic acid, 3, 4‐dihydroxybenzoic acid, chlorogenic acid, ferulic acid, ellagic acid, *p*‐coumaric acid, sinapic acid, quercetin 3‐D‐galactoside, quercetin 3‐glucoside, luteolin, pelargonidin‐3‐*O*‐glucoside, pelargonidin chloride, delphinidin‐3‐*O*‐β‐D‐glucoside, malvidin chloride, quercetin, rutin trihydrate, rutin (quercetin 3‐rutinoside), kaempferol, naringenin, chrysin, and myricetin] were HPLC‐grade and purchased from Sigma‐Aldrich (Darmstadt, Germany). All other chemicals and reagents used in the experiments were of analytical grade.

### 2.3 Infusion of Fresh‐Cut Apple in the Hibiscus Extract

For the extract preparation and infusion, the methods described by Stan (Boldea), Aprodu, et al. (2023) and Stan (Boldea), Nistor, et al. (2023) were used. Briefly, in order to obtain the extract, 10 g of hibiscus calyx was added to 1000 mL of boiling water and infused at 100°C for 15 min. The apples (about 1 kg) were washed with tap water, blotted gently with tissue paper to remove the excess water, and sliced using a food slicer (Gorenje R707A, Velenje, Slovenia) at a 3‐mm thickness. The slices were divided into two experimental groups, including the control (code C_temperature_CD and IR, respectively) and the infused samples (code C_temperature_CDH and IRH, respectively). The apple slices were immersed in the hibiscus aqueous extract and maintained at 70°C for half an hour, whereas the control pieces were treated under the same conditions but replacing the extract with distilled water. After half an hour, the pieces were removed from the solutions, allowed to drain at room temperature for 5 min, and subjected to drying.

### 2.4 Experimental Procedure

Two drying methods were used in the experiment: CD and IR. Approximately 250 ± 5 g of sliced apple was dried in each experiment. The drying of apple slices was performed using an infrared‐convective dryer equipped with 5 perforated trays (Concept SO4000 Infra 500 W, Chocen, Czech Republic). The apple slices were dried at constant temperatures of 50°C, 60°C, and 70°C using an air velocity of 1.2 ± 0.1 m/s measured with a hotwire thermo‐anemometer VT 115 (Kimo Instruments, Millgrove, Ontario, Canada) and a relative humidity of 12.2% ± 1.5%. The drying agent used to obtain apple snacks consisted mainly of hot air, providing the energy required for water evaporation and removing water vapor out from the dryer. Relative humidity was measured with a thermo hygrometer EE33 Series, fitted with a sensing probe (E + E Electronik Ges.m.b.H. Engerwitzdorf, Austria). At every 30 min, the apple slice samples were weighed using a digital balance Precisa EP–125SM (Precisa, Iasi, Romania) with an accuracy of 0.01 g. The drying process was stopped when the apple slice samples reached constant weight (equilibrium condition). After drying, the samples were taken to the desiccator for about 30 min to cool at room temperature (22°C ± 2°C) and then packed in a paper bag and stored in a desiccator in the dark until they were analyzed.

### 2.5 Mathematical Modeling of Drying Curves

The moisture ratio (MR) and drying rate (DR) were calculated based on Equation (1) (Veleșcu et al. 2023):

(1) $$\mathrm{MR}=\frac{M_{t}-M_{e}}{M_{0}-M_{e}}$$

where MR is the moisture ratio (dimensionless); *M*_t_, *M*_0_, and *M*_e_ are moisture content at time *t* (kg·kg^1^), at initial and equilibrium moisture content (kg/kg).

The Equation (2) was used to compute the DR (Veleșcu et al. 2023):

(2) $$\mathrm{DR}=\frac{M_{t+\mathrm{dt}}-M_{t}}{d_{t}}$$

where *M*_t + dt_ is the moisture content at time t + *d*_t_ (kg/kg) and *d*_t_ is the time interval between two consecutive moisture measurements (min). Table S1 summarizes the mathematical models applied for kinetic modeling of the drying curves (Lewis 1921; Page 1949; Henderson and Pabis 1962; Meisami‐asl et al. 2009). A nonlinear regression analysis (CurveExpert Professional software version 2.7.3—free trial) was used to estimate the coefficients of the mathematical models. The best fitted mathematical model used to describe the drying kinetic parameters was selected based on the highest value of the coefficient of correlation (*R* ^*2*^) and the lowest value of the sum of the residual squares (SSR).

### 2.6 Moisture Effective Diffusivity (*D*_eff_) and Activation Energy (*E*_a_)

The effective diffusivity of dried apple sliced samples (*D*_eff_) was estimated based on Equation (3), which describes Fick's second law of diffusion, describing the drying process and the transport of moisture inside biological materials (Demiray et al. 2023).

(3) $$\mathrm{MR}=\frac{8}{\pi^{2}}\sum_{n=0}^{\infty }\frac{1}{{\left(2n+1\right)}^{2}}\exp\left[-{\left(2n+1\right)}^{2}\times \frac{\pi^{2}}{4}\times \frac{D_{\mathrm{eff}}\times t}{L^{2}}\right]$$

where MR is the moisture ratio, *t* is the time (s), *L* is the thickness of samples (m) and *D*_eff_ is the effective diffusivity (m^2^/s).

In the case of prolonged drying times, Equation (3) is expressed in a logarithmic form (Equation 4):

(4) $$\text{lnMR}=\ln\left(\frac{8}{\pi^{2}}\right)-\left(\pi^{2}\times \frac{D_{\mathrm{eff}}}{4\times L^{2}}\times t\right)$$

The *D*_eff_ was calculated by plotting ln MR against drying time, by applying Equation (5):

(5) $$\text{Slope}=\frac{\pi^{2}\times D_{\mathrm{eff}}}{4\times L^{2}}$$

The relation between *D*_eff_ and temperature was assumed to be the Arrhenius equation, and *E*_a_ was calculated using Equation (6):

(6) $$D_{\mathrm{eff}}=D_{0}\exp\left(-\frac{E_{a}}{R\times T}\right)$$

where *D*_0_ is the pre‐exponential factor of the Arrhenius equation (m^2^/s), *E*_a_ is the activation energy (Kj/mol), *T* is the absolute temperature (K), and *R* is the universal gas constant (*R* = 8.31451 J/mol/K). A plot of ln (*D*_eff_) against 1/*T* gives a straight line with a slope of *E*_a_/*R*.

### 2.7 Phytochemical Extraction

For polyphenolic extraction from apple snacks, 1 g of each sample was crushed and then mixed with 9 mL of ethanol solution (70%) and allowed to extract into an ultrasonic water bath, at a constant temperature of 30°C for 30 min. Further, the samples were centrifuged at 7000 × g at 4°C for 10 min, whereas the supernatants were used to analyze the polyphenols by the chromatographic method and antioxidant activity.

### 2.8 Chromatographic Analysis of Polyphenols

The chromatographic analysis in order to characterize the entire polyphenolic profile was undertaken on an Agilent 1200 HPLC system (Agilent Technologies, Santa Clara, CA, USA) and was based on an elution with 10% formic acid (v/v, solvent A) and 100% methanol (v/v, solvent B) using a a C18 Synergi 4u Fusion‐RP 80A column, 150 mm × 4.6 mm, and particle size of 4 μm (Phenomenex, California, USA). The flow rate was 1 mL/min, at 30°C, with an injection volume of 10 μL of sample, the detection taking place at the 280 and 320 nm wavelengths. The compounds were identified and also quantified based on the available standards and the data reported in the scientific literature (Anghel et al. 2023).

### 2.9 Antioxidant Activity

The antioxidant capacity of the apple snacks extracts was determined at the ability to scavenge the DPPH radical. For each sample, volumes of 100 μL were mixed with 3900 μL of DPPH methanolic solution (0.1 M), followed by incubation at room temperature for 30 min in the dark (Anghel et al. 2023). The absorbance was measured at 515 nm against blank UV–visible spectrophotometer (Biochrom Libra S22 UV/Vis, Cambridge, UK). All the experiments were performed in triplicate. The antioxidant activity was expressed as mMol Trolox/g dry weight (DW) using a Trolox‐based calibration curve (*y* = 0.45*x* − 0.0075, R2 = 0.993).

### 2.10 Texture Analysis

For texture measurements, a CT3 Texture (Brookfield Ametek, USA) analyzer equipped with a 1000 g load cell was used. The apple snacks were placed on a perforated platform, and a 4 mm diameter metallic cylinder was used to determine firmness and crispness. Pretest and posttest speed was set at 2 mm/s, test speed was 0.5 mm/s, and the trigger load was 0.067 N. The firmness was expressed as the maximum force required to break the snacks, and the crispness was determined as the number of peaks in the deformation‐force diagram (Xiao et al. 2018). Five measurements were performed for each sample.

### 2.11 Colorimetric Parameters

The color parameters (L*, a*, b*, C*, and h*) of fresh and dried apple snacks were measured with an NR110 precision colorimeter (Shenzhen 3nh Technology Co., Guangdong, China). The total color change (Δ*E*), browning index (*BI*), whiteness index (WI) and yellowness index (YI) of the fresh and dried samples were calculated using Equations ((7), (8), (9), (10), (11)) (Duan et al. 2017; Dajbych et al. 2023).

(7) $$∆E=\sqrt{{\left(L_{0}^{*}-L^{*}\right)}^{2}+{\left(a_{0}^{*}-a^{*}\right)}^{2}+{\left(b_{0}^{*}-b^{*}\right)}^{2}}$$

(8) $$\mathrm{BI}=\frac{\left[100\times \left(x-0.31\right)\right]}{0.17}$$

(9) $$x=\frac{\left(a^{*}+1.75\times L^{*}\right)}{\left(5.645\times L^{*}+a^{*}-3.012\times b^{*}\right)}$$

(10) $$\mathrm{WI}=100-\sqrt{{\left(100-L^{*}\right)}^{2}+a^{*2}+b^{*2}}$$

(11) $$\mathrm{YI}=\frac{142.86\times b^{*}}{L^{*}}$$

where *L*_0_*, *a*_0_,* and *b*_0_* are the color parameters of the fresh samples, L*, a*, and b* represent the corresponding color parameters of dried samples, L* (lightness/darkness), a* (redness: green to red), and b* (yellowness: blue to yellow). All the experiments were performed in triplicate.

### 2.12 RR

Dried apple snacks (7 ± 0.5 g) were immersed in 200 mL of distilled water in a 250 mL beaker and rehydrated at 40°C. Samples were weighed at 5 min intervals. Then, the dried apple snacks were taken out, and the surface water was carefully absorbed with paper towels for 60 s and then weighed. After weighing, the samples were immediately returned to the same beaker with distilled water. This procedure was repeated until constant weight was obtained (at least two consecutive weighing). Equation (12) was used to calculate the RR of the samples (Zhu et al. 2022).

(12) $$\mathrm{RR}=\frac{m_{2}}{m_{1}}$$

where *m*_1_ is the weight of dried apple snacks before rehydration (g) and *m*_2_ is the weight of dried apple snacks after rehydration (g). All the experiments were performed in triplicate.

### 2.13 Shrinkage Ratio

The SR of the apple snacks was calculated using Equations ((13), (14), (15)) (Dajbych et al. 2023).

(13) $$\mathrm{SR}=\frac{\left(V_{i}-V_{f}\right)}{V_{i}}\times 100$$

where *V*_i_ is the initial volume of the fresh apple sample (m^3^) and *V*_f_ is the final volume of the dried apple sample (m^3^).

(14) $$V_{i}=\pi \times {\left(\frac{D_{i}}{2}\right)}^{2}\times t_{i}$$

(15) $$V_{f}=\pi \times {\left(\frac{D_{f}}{2}\right)}^{2}\times t_{f}$$

where *D*_i_, *D*_f_, *t*_i_, and *t*_f_ are the initial and final diameters (mm) and thicknesses (mm) of the samples. Diameters and thicknesses of the samples were measured using a digital caliper (Burg Wachter Digital Caliper Precise PS 7215, 150 × 0.01 mm) with an accuracy of 0.01 mm. All the experiments were performed in triplicate.

### 2.14 Surface Area (A)

The fresh and dried apple snacks surface area *A* (mm^2^) was calculated using Equation (16) (Dajbych et al. 2023).

(16) $$A=2\times \pi \times r\times \left(r+h\right)$$

where *r* is the radius (mm) and *h* is the thickness (mm) of the fresh and dried apple snack samples. All the experiments were performed in triplicate.

### 2.15 Sensory Analysis

The scoring method with different levels of perception, ranging from 1 (very low) to 7 (very high), described by Stan (Boldea), Nistor, et al. (2023) was used for the sensory evaluation of the apple snacks. Before analysis, 10 panelists with ages between 23 and 35 years were trained on the apple snack sensory‐relevant characteristics, including color, appearance, flavor, taste, crispness, hardness, and overall acceptability. The samples were tempered at 22°C ± 1°C for 1 h, in a controlled atmosphere and displayed in clear, coded glass jars. Prior to and between each analysis, the panelists were asked to clean their oral cavity with water. The acceptability index (AI) was calculated based on Equation (17):

(17) $$\text{Acceptability index}\;\left(\%\right)=\frac{A}{B}$$

Where *A* is the average of the hedonic values obtained for an individual attribute, and *B* is the maximum hedonic value attributed to the same attribute.

### 2.16 Statistical Analysis

All the experiments were performed in triplicates, except for HPLC, which was performed in duplicates. All the data expressed on a dry basis are presented as mean ± standard error. One‐way analysis of variance (ANOVA) was used to analyze statistically significant differences among the results, followed by the Tukey test at a significance level of 5% (*p* < 0.05). The statistical software package Minitab version 19 (Romsym Data, Bucharest, Romania) was used for the analysis.

## 3 Results and Discussion

### 3.1 Analysis of Drying Curves and Mathematical Modeling

**FIGURE 1:**
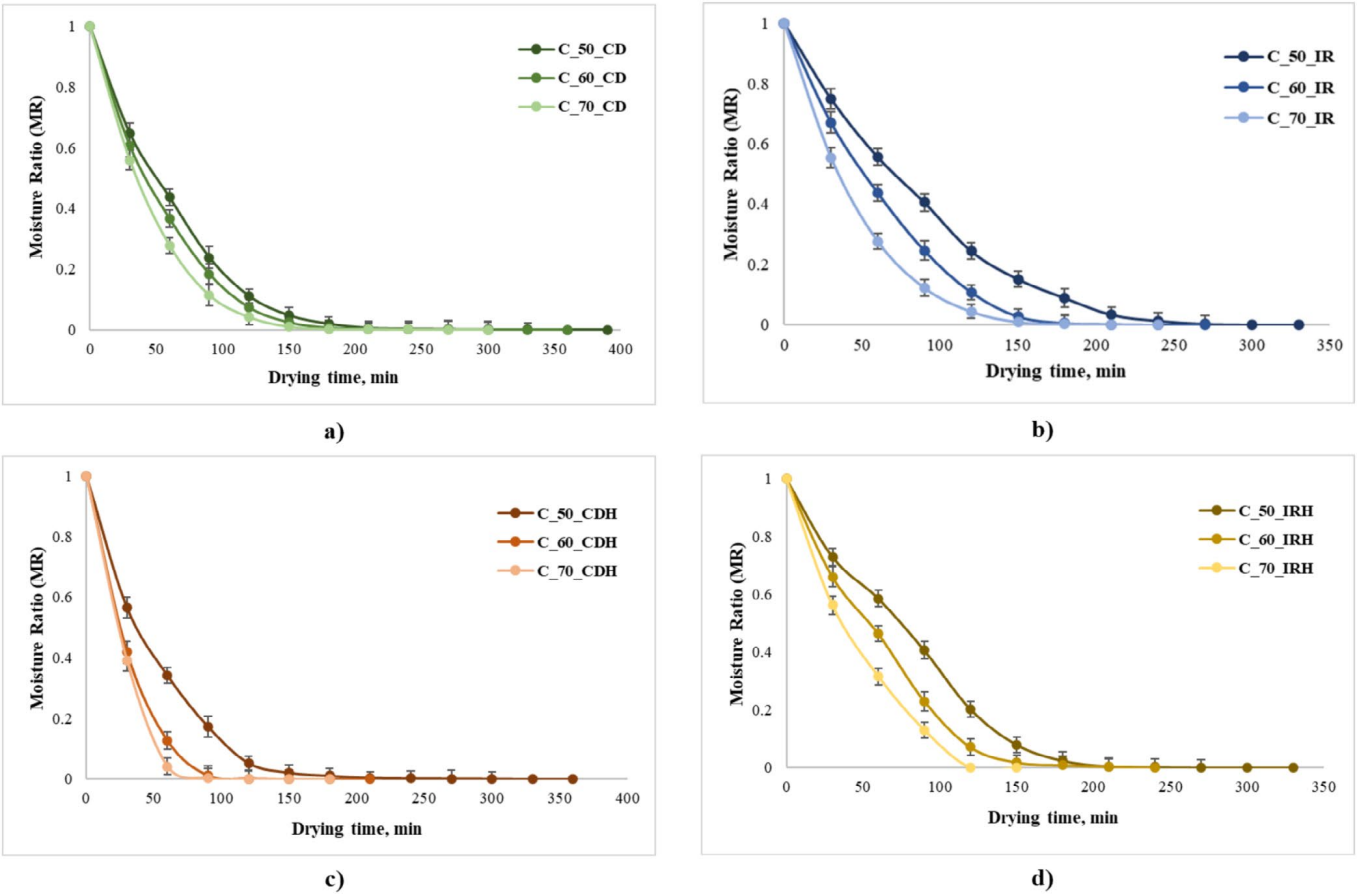
Drying kinetics curves of apple slices under different drying methods and temperatures (a—apple slices convective air‐dried; b—apple slices infrared‐dried; c—apple slices infused with the *Hibiscus sabdariffa* extract convective air‐dried; d—apple slices infused with the *Hibiscus sabdariffa* extract infrared‐dried).

The effect of temperature and the type of drying on MR change of fresh and *Hibiscus sabdariffa* extract‐infused apple is shown in Figure 1. For both CD and IR, the increase in temperatures resulted in a reduction of the drying times, leading to a continuous decrease in MR values, as a function of drying time. A possible explanation for the observed correlations is related to the heat transfer rate among the apple slices and the drying agent inside the drying equipment, which increased with temperature, generating an acceleration of moisture diffusion from inside to the surface, favoring evaporation. Our results are in good agreement with Demiray et al. (2023) and Huang et al. (2022), who studied the effect of drying temperatures, air velocities, and infrared radiation on apple slices. The effect of increased cell water removal is due to the increase in the drying temperatures, simultaneously with the increase in the water vapor pressure from apple slices. As can be observed from Figure 1, the drying period varied between 180 and 390 min for CD and between 150 and 330 min for IR. Compared to CD, the dehydration time of the IR drying was about 25% lower when compared with control and with approximately 17% for the hibiscus extract‐infused samples.

**FIGURE 2:**
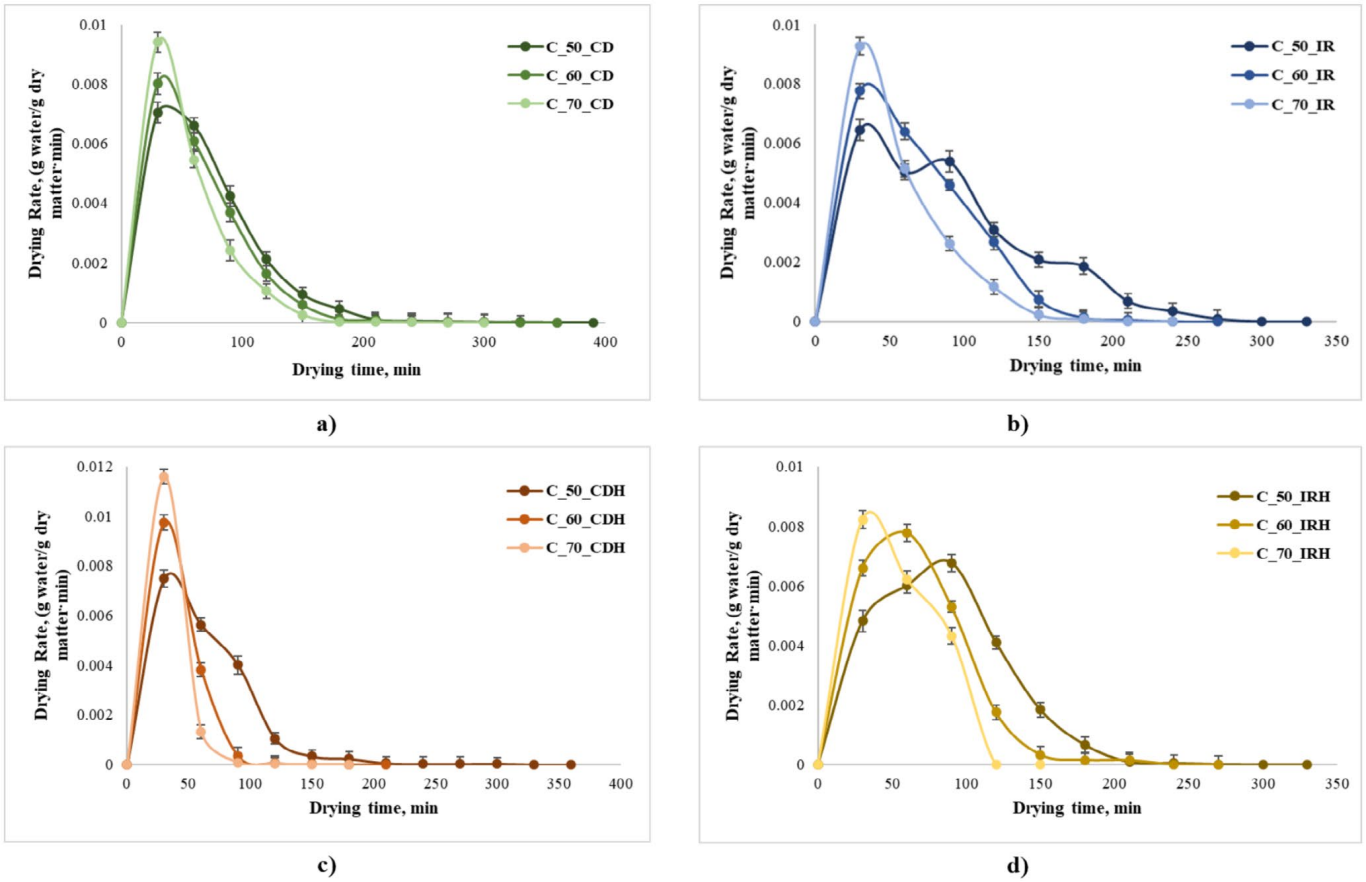
Changes in the drying rate of apple slices versus drying time (a—apple slices convective air‐dried; b—apple slices infrared‐dried; c—apple slices infused with the *Hibiscus sabdariffa* extract convective air‐dried; d—apple slices infused with the *Hibiscus sabdariffa* extract infrared‐dried).

The DRs decrease continuously with drying time, as shown in Figure 2. For both drying methods and all samples, a constant rate of drying period was not observed. From Figure 2 two distinct drying periods can be observed. In the first period, known as the warming‐up stage, the DRs reached the maximum values for both drying methods after 30 min (0.007–0.0115 g water/g DW for CD and 0.0048–0.0092 g water/g DW for IR drying), favored by the highest moisture content of the samples at the beginning of drying.

The second drying period takes more slowly, given the slow rate for the reduction of moisture content in the samples. Therefore, as it can be observed, significantly low DR (10^2^–10^4^ times lower) were found in the second stage, such as between 0.0000075 and 0.000038 g water/g DW for CD and 0.000016 and 0.0043 g water/g DW for IR drying. This aspect can be explained by the prolonged drying time, which led to a decrease in porosity due to higher shrinkage, leading to a further decrease in DRs. Similar results were found by Onwude et al. (2018) for mid‐IR of sweet potatoes and Abdelbasset et al. (2022), who studied the effect of infrared radiation at different temperatures on pumpkin drying.

The kinetic parameters resulted from mathematical modeling for both CD and IR drying at different temperatures, together with the statistical parameters (*R* ^*2*^ and *SSR*), are presented in Table S2. The mathematical model with the highest *R* ^*2*^ value and the lowest *SSR* values was selected as the adequate model for describing the CD and IR drying processes of the samples.

The Page model has the best match for all drying temperatures and methods, with the highest *R* ^2^ values varying from 0.990 to 0.996 and the lowest SSR values varying from 0.0037 to 0.149 when compared to other used models. Similar results were obtained by Dajbych et al. (2023) for infrared and hot air drying of Red Delicious apple slices, Salehi and Satorabi (2021) for IR of apple slices covered with basil seed and xanthan gums, and Demiray et al. (2023) for CD of Granny Smith apple slices.

### 3.2 *D*_eff_ and *E*_a_

The values of effective moisture diffusivity (*D*_eff_) and activation energy (*E*_a_) under different drying conditions are shown in Table 1. It was found that *D*_eff_ values of the apple samples dried with the CD varied between 2.31 × 10^−8^ and 3.16 × 10^−8^ m^2^/s for the control group and from 2.22 × 10^−8^ to 6.18 × 10^−8^ m^2^/s for the samples infused with hibiscus extract, respectively. For the samples dried with the IR method, *D*_eff_ values varied between 1.85 × 10^−8^ and 3.05 × 10^−8^ m^2^/s for the control group and from 2.64 × 10^−8^ to 3.25 × 10^−8^ m^2^/s for the infused samples, respectively. As expected, the *D*_eff_ values increased with an increase in drying temperatures (Maleki et al. 2020). However, it can be observed that *D*_eff_ values for CD at 70°C are two times higher when compared with IR at the same temperature. The obtained results highlight the accelerated diffusion of water from slices with the increasing temperature. The corresponding *D*_eff_ values were in the range of 10^−12^ to 10^−8^, which are usually found for biological materials (Jha and Sit 2020). Similar variations were also observed during drying by Rayaguru and Routray (2012) for stone apple slices that were hot air‐dried and by Uğurlu et al. (2023) for apple slices dried with an infrared vacuum dryer. The activation energy (*E*_a_) values of apple slices dried with CD were between 14.31 and 47.23 Kj/mol, while for the IR dried samples, they were between 9.95 and 23.02 Kj/mol. Similar values were reported for different fruits and vegetables: 17.77–25.41 Kj/mol for apple slices (Beigi 2016) and 10.59–54.93 Kj/mol for yam slices (Ojediran et al. 2020). According to Srikanth et al. (2019), the differences in the *E*_a_ values may be due to some factors such asripening degree, tissue structure and components, variety, texture, size, and surface area.

**TABLE 1:** Effective moisture diffusivity (*D*_eff_) and activation energy (*E*_a_) of apple slice samples.

Sample code	Drying method	*t*, °C	*D*_eff_ × 10^−8^, m^2^ s^−1^	*R* ^2^	*E*_a_, kJ·mol^−1^	*R* ^2^
C_50_CD	Convective air drying	50	2.31	0.9896	14.31	0.9943
C_60_CD		60	2.78	0.9860		
C_70_CD		70	3.16	0.9808		
C_50_CDH		50	2.22	0.9856	47.23	0.9398
C_60_CDH		60	4.73	0.9828		
C_70_CDH		70	6.18	0.9799		
C_50_IR	Infrared drying	50	1.85	0.9236	23.02	0.9925
C_60_IR		60	2.81	0.9408		
C_70_IR		70	3.05	0.9695		
C_50_IRH		50	2.64	0.9362	9.95	0.9980
C_60_IRH		60	2.90	0.9707		
C_70_IRH		70	3.25	0.9715		

Abbreviations: C_*temperature*_CD, apple slices dried by convective air drying at 50°C, 60°C, and 70°C; C_*temperature*_CDH, apple slices infused in the *Hibiscus sabdariffa* extract by convective air drying at 50°C, 60°C, and 70°C; C_*temperature*_IR, apple slices dried by infrared drying at 50°C, 60°C, and 70°C; C_*temperature*_IRH—apple slices infused in the *Hibiscus sabdariffa* extract by infrared drying at 50°, 60°, and 70°.

### 3.3 Color Parameters of Apple Snacks

Color parameters are important quality parameters of dried fruits and are associated with the acceptability of the product since a product is better accepted by consumers if its visual characteristics (color and size) are close to unprocessed fruits (Ghinea et al. 2022). Table 2 presents the results of color parameters of the apple snacks resulting from different drying methods. As expected, the color parameters changed during the drying process. The values of the L* parameter in the case of dried samples are higher compared with the *L*_*0*_ * values, indicating a lighter color for apple snacks compared with fresh apples. When drying, the lightness (L*) increased for all samples with the maximum values of 59.2% and 68.4% for the CD and IR, respectively. The drying process also led to a decrease in the red/green ratio (a*). The values of this color parameter decreased with the increasing drying temperature. The reduction of a* value of dried samples can be due to the appearance of Maillard products (Ìzli et al. 2014). Based on a* values, it seems that the anthocyanins from the flowers of *Hibiscus sabdariffa* are responsible for the pleasant red color of the infused apple slices (Hernández‐Carranza et al. 2022). The temperature of the drying agent has an influence on the yellow/blue parameter (b*). An increase in the b* parameter was observed only for control samples (for both drying methods) compared with the fresh sample. In the case of samples infused with hibiscus extract, the values of this color parameter recorded a slight decrease. This may occur due to the changes in the structural properties of the apple slice tissue.

**TABLE 2:** Color parameters of fresh and dried apple slice samples.

Color parameters/Sample code*	*L* *	*a* *	*b* *	ΔE	*C* *	*h* *	BI	WI	YI
*Fresh apple slices*									
C_0_	37.21 ± 0.82^B^	19.19 ± 0.36^B^	19.69 ± 0.37^A^	—	27.67 ± 0.29^A^	46.08 ± 0.47^B^	109.54 ± 2.19^A^	31.45 ± 0.76^A^	75.63 ± 0.83^A^
CH_0_	52.37 ± 1.62^A^	35.52 ± 0.86^A^	18.61 ± 0.35^A^	—	18.92 ± 0.39^B^	79.61 ± 0.76^A^	89.58 ± 1.39^B^	—	—
*Convective drying*									
C_50_CD	43.55 ± 1.15^Cb^	12.18 ± 0.91^Ca^	29.58 ± 0.51^Aa^	13.71 ± 1.28^Cb^	32.01 ± 0.20^Aa^	68.28 ± 1.20^Bb^	125.53 ± 3.35^Aa^	35.11 ± 0.92^Cb^	97.06 ± 0.96^Aa^
C_60_CD	59.25 ± 1.15^Ab^	9.08 ± 0.56^Db^	28.44 ± 1.05^Ab^	25.79 ± 1.24^Ab^	29.41 ± 1.29^Bb^	72.34 ± 1.19^Aa^	75.00 ± 5.23^Cb^	49.48 ± 1.51^Ab^	68.62 ± 3.72^Bb^
C_70_CD	53.50 ± 0.69^Bb^	6.90 ± 0.59^Db^	22.93 ± 0.86^Bb^	20.67 ± 1.54^Bb^	23.95 ± 0.98^Cb^	73.26 ± 0.86^Aa^	64.28 ± 4.43^Da^	47.69 ± 1.04^Bb^	61.26 ± 3.01^Ca^
C_50_CDH	38.05 ± 0.89^Da^	26.18 ± 0.72^Aa^	17.24 ± 1.14^Ca^	17.18 ± 0.64^BCb^	31.35 ± 1.18^Aa^	33.33 ± 1.17^Ca^	106.27 ± 3.83^Bb^	—	—
C_60_CDH	42.73 ± 1.17^Cb^	21.12 ± 1.14^Ba^	15.55 ± 0.69^Ca^	17.60 ± 1.23^BCa^	26.23 ± 1.32^BCa^	36.37 ± 0.26^Ca^	79.45 ± 4.23^Ca^	—	—
C_70_CDH	37.09 ± 0.86^Db^	23.16 ± 1.10^Ba^	16.36 ± 0.62^Ca^	19.80 ± 0.71^Ba^	28.37 ± 0.55^Ba^	37.59 ± 0.43^Ca^	100.42 ± 3.80^Ba^	—	—
*IR drying*									
C_50_IR	53.82 ± 0.93^Ba^	8.26 ± 0.53^Db^	28.08 ± 0.42^Bb^	21.59 ± 0.90^Ba^	29.27 ± 0.53^BCb^	73.95 ± 0.31^Aa^	82.53 ± 0.80^Cb^	45.32 ± 0.54^Ca^	74.54 ± 0.75^Ab^
C_60_IR	62.66 ± 1.24^Aa^	10.28 ± 0.76^Ca^	30.75 ± 1.07^Aa^	29.16 ± 0.97^Aa^	32.37 ± 1.32^Aa^	71.82 ± 1.09^Bb^	77.63 ± 3.34^Ca^	50.53 ± 0.84^Ba^	70.10 ± 1.91^Ba^
C_70_IR	62.07 ± 1.47^Aa^	7.60 ± 0.67^Da^	26.62 ± 0.59^Ba^	28.29 ± 1.47^Aa^	27.67 ± 0.54^CDa^	73.61 ± 0.68^Aa^	63.76 ± 1.82^Db^	53.03 ± 1.02^Aa^	61.27 ± 0.80^Ca^
C_50_IRH	35.69 ± 0.76^Db^	26.00 ± 1.13^Aa^	16.54 ± 1.06^Cb^	19.36 ± 1.58^Ca^	30.82 ± 1.24^ABb^	32.48 ± 1.72^Cb^	110.68 ± 6.77^Aa^	—	—
C_60_IRH	44.13 ± 1.26^Ca^	21.27 ± 0.90^Ba^	14.60 ± 0.96^Db^	16.97 ± 2.02^Db^	25.80 ± 1.20^Db^	34.46 ± 1.15^Cb^	73.59 ± 4.01^CDb^	—	—
C_70_IRH	38.03 ± 1.79^Da^	22.20 ± 1.01^Bb^	16.37 ± 0.33^Ca^	19.72 ± 1.41^Ca^	27.59 ± 1.01^CDb^	36.42 ± 0.70^Cb^	96.18 ± 6.19^Bb^	—	—

Abbreviations: *a* *, intensity of green (−60 < *a* * < 0) or red (0 < *a* * < +60); *b* *, intensity of blue (−60 < *b* * < 0) or yellow (0 < *b* * < +60); BI, browning index; *C* *, color intensity; *h* *, visual color appearance; *L* *, lightness/darkness (*L* * = 100 white; *L* * = 0 black); WI, whiteness index; YI, yellowness index; Δ*E*, total color difference.

* C_0_: Fresh apple slices sample, CH_0_: fresh apple slices sample infused with the *Hibiscus sabdariffa* extract, C_CD: apple slices dried by the convection method, C_CDH: apple slices infused with the *Hibiscus sabdariffa* extract dried by the convection method, C_IR: apple slices dried by the infrared (IR) method, C_IRH: apple slices infused with the *Hibiscus sabdariffa* extract dried by the IR method. Within a set of experiments, means at different drying treatments, on the same column that do not share a superscript letter, are significantly different at *p* < 0.05; within a set of experiments, means at the same drying temperature, on the same column that do not share a lowercase letter, are significantly different at *p* < 0.05 based on the Tukey test.

Another key factor, correlated with the product quality during drying, is the total color change (*∆E*), a parameter that highlights the color changes that occur during drying compared to the fresh product. The different values are suggested by the complex airflow field around the slice, which induced a spatial variation in convective heat/mass transfer coefficients over the half‐circular apple fruit surface, as explained by Defraeye and Radu (2018), for the convective hot air drying environment. In our study, the *∆E* values were quite similar and varied from 13.71 ± 1.28 to 25.79 ± 1.24 for CD samples and from 16.97 ± 2.02 to 29.16 ± 0.97 for apple slices IR‐dried. According to Kidoń and Grabowska (2021), the values of *∆E* higher than 1 confirm that there are visible differences to the human eye. The temperature and the drying method had an important effect on the color intensity (C*), color tone (h*), whiteness index (WI), and the yellowness index (YI) of the apple snacks (Table 2). Another important indicator for the browning reaction is the browning index (BI), which is a significant factor in enzymatic and non‐enzymatic browning processes. The value of BI for fresh apple slices was 109.54 ± 2.19. In general, the inhibitory effect of *Hibiscus* extract is visible for the fresh sample, with a decrease in BI from 109.54 ± 2.19 in fresh‐cut apples to 89.58 ± 1.39 for the infused samples. Based on the obtained data, it is evident that the air temperature, velocity, and drying time have an influence on the dried apple slices color parameters. When drying, it can be observed from Table 2 that, in the case of CD, when drying at 50°C, the BI increases, due to the enzymatic browning taking place during the cutting and preparation of the samples. In control samples, the BI decreases significantly with the increasing temperature, due to the partial inactivation of PPO and POD. In the case of infused samples, the BI decreases when drying between 50° and 60° and increases at 70°C, due probably to the effect of temperature on anthocyanins, inducing polymerization and/or copolymerization, thus favoring the non‐enzymatic browning at higher temperatures.

### 3.4 Textural Analysis

Texture is one of the most important characteristics for snacks due to the special sensation produced by their disintegration during consumption. This is associated with firmness and crispness, which are presented in Table 3. Both parameters are influenced by the drying treatment and by the infusion process. Therefore, for CD, a significant (*p* < 0.05) decrease in firmness of apple snack without hibiscus was found with the increasing temperature and from 6.21 ± 0.44 N when drying at 50°C to 4.74 ± 0.57 N at 70°C. The infused dried samples showed a significantly lower firmness (*p* < 0.05), which decreased up to 2.70 ± 0.40 N when drying at 70°C (Table 3). For IR drying, the corresponding values at all temperatures were lower when compared with CD. Moreover, the crispness values increased with the increasing temperature for all the dried samples. The CD‐treated samples showed significantly higher (*p* < 0.05) values for infused slices in hibiscus extract, from 9.40 ± 0.55 to 17.60 ± 0.55 when drying at 70°C, whereas the IR control and infused samples showed significantly higher values when compared with CD. The highest value for crispness reached 34.60 ± 1.14 (Table 3). Xiao et al. (2018) explained this behavior as a result of a limited rate of water migration to the surface of the tissue, leading to a hard and dry crust at the surface area. Therefore, the infusion of the apple slices with *Hibiscus sabdariffa* extract determined a decrease in firmness of 10%–30% and an increase in crispness of 50%–70%.

**TABLE 3:** Texture properties of apple snacks.

Type of drying	Temperature, °C	Firmness, N		Crispness	
		Apple snacks	Apple snacks infused with the *Hibiscus sabdariffa* extract	Apple snacks	Apple snacks infused with the *Hibiscus sabdariffa* extract
CD	50	6.21 ± 0.44^Aa^	5.05 ± 0.87^Aa^	4.60 ± 0.55^Cb^	15.00 ± 1.41^Bb^
	60	5.57 ± 0.20^ABa^	3.94 ± 0.55^ABa^	6.00 ± 0.71^Bb^	16.20 ± 0.84^ABb^
	70	4.74 ± 0.57^B^	2.70 ± 0.40^Ba^	9.40 ± 0.55^Ab^	17.60 ± 0.55^Ab^
IR	50	5.35 ± 0.48^Aa^	4.86 ± 0.39^Aa^	14.00 ± 1.00^Ba^	19.40 ± 0.54^Ca^
	60	4.90 ± 0.62^ABa^	3.06 ± 0.72^ABa^	15.80 ± 0.84^ABa^	29.20 ± 1.30^Ba^
	70	3.99 ± 0.25^Ba^	2.13 ± 0.51^Ba^	17.80 ± 0.84^Aa^	34.60 ± 1.14^Aa^

*Note:* Within a set of experiments means at different drying treatments, on the same column that do not share a superscript letter, are significantly different at *p* < 0.05; within a set of experiments means for control and infused apple snack, on the same row that do not share a lowerscript letter, are significantly different at *p* < 0.05 based on the Tukey test.

### 3.5 Rehydration Ratio, SR, and Surface Area

For dried products, a comprehensive quality parameter is represented by RR. This parameter is influenced by drying conditions and product composition (Shewale and Hebbar 2017). A high value of the hydration capacity for the dried products is strongly correlated with a greater ability to resorb water, which denotes minor damage in the product structure due to drying (Yi et al. 2016). Figure S1 shows the results for RR of the apple slices dried at different temperatures and drying methods. The RR of apple slices samples increased with increasing drying temperatures. Within the first 5 min, the RR values of all apple slices samples (control and infused samples) were higher for both drying methods. The RR of IR dried samples reached the maximum value (3.75 ± 0.16 for control samples and 4.88 ± 0.14 for infused samples) after 40 min compared with CD samples, where the RR reached the maximum value after 50 min (3.71 ± 0.13 for control samples) and 40 min (5.01 ± 0.14 for samples infused with hibiscus extract). The higher RR values of infused samples may be explained by the presence of the bioactive compounds from *Hibiscus sabdariffa* calyx aqueous extract that probably created a barrier to water penetration, thereby improving the RR. Similar results were obtained by Zhu et al. (2022) on apple snacks dried with different drying methods.

The SR of dried apple slice samples under different drying conditions is presented in Figure S2. The SR of apple slice samples varied with the increase in drying temperature and time. The maximum value of SR for both drying methods was found at a temperature of 70°C (84.11% ± 1.41% for CD and 86.13% ± 1.61% for IR drying) for the control samples, while for the slice infused with *Hibiscus sabdariffa* extract, the maximum value of SR was established at 50°C (85.68% ± 1.68% for CD and 81.13% ± 1.81% for IR drying).

The high drying temperature increases the moisture diffusion rate, enhancing the texture and microstructure of the apple slice samples. The SR values obtained in the present research were in the range of 70%–90%, values usually characteristic of fruits and vegetables (Alaei and Chayjan 2014). Similar results were found by Alaei et al. (2018) for quince slices using infrared vacuum drying and Wang et al. (2018) for lemon slices using pulsed vacuum drying.

The surface area was measured to identify the changes in size depending on the different drying temperatures and methods. The surface area of the dried apple slices, for both drying methods, recorded a slight decrease. The values of the final surface area of the dried apple sliced samples for CD ranged from 0.003404 to 0.006007 m^2^, while for IR drying, the surface area values ranged from 0.003914 to 0.006121 m^2^. Çetin et al. (2019) indicated similar results for the Red Chief variety apple using different drying conditions (drying temperature and time, slice thickness).

### 3.6 Sensorial Analysis

The sensorial analysis allowed us to evaluate the following sensory parameters: appearance, color, taste, flavor, hardness, crispness, and overall acceptability. From Table 4 it can be observed that for appearance, no statistical differences were observed (*p* < 0.05), except for the CD control and infused samples dried at 50°C. The color received the highest scores for the samples infused with hibiscus extract, due to the coloring effect of anthocyanins. Taste was appreciated with scores around 6, whereas IR‐dried samples infused with hibiscus received higher scores. According to the textural analysis, the panelists evaluated with lower values the hardness of infused samples, when compared with the control, whereas crispness received higher scores for the infused samples, dried by both methods at 70°C. The highest scores for overall acceptability were found for the samples dried at 70°C. Given the above‐mentioned results, control and infused samples dried at 70°C with both methods were chosen for chromatographic profiling.

**TABLE 4:** Sensorial analysis of fresh and dried apple slice samples.

Characteristics/Sample code*	Appearance	Color	Taste	Flavor	Hardness	Crispness	Overall acceptability
*Fresh apple slices*							
C_0_	6.40 ± 0.52^A^	4.90 ± 0.57^A^	6.00 ± 0.67^A^	5.60 ± 0.70^A^	4.80 ± 0.42^A^	2.00 ± 0.67^A^	5.90 ± 0.57^A^
CH_0_	6.40 ± 0.52^A^	5.90 ± 0.57^A^	6.20 ± 0.42^A^	5.60 ± 0.70^A^	3.00 ± 0.47^B^	1.80 ± 0.63^A^	3.00 ± 0.67^B^
*Convective drying*							
C_50_CD	6.40 ± 0.52^Aa^	5.70 ± 0.67^ABa^	6.40 ± 0.52^Aa^	6.20 ± 0.63^Aa^	4.80 ± 0.42^Aa^	3.40 ± 0.52^Ba^	5.60 ± 0.52^Ba^
C_60_CD	6.40 ± 0.52^Aa^	5.10 ± 0.32^Ba^	6.40 ± 0.52^Aa^	6.10 ± 0.88^ABa^	5.20 ± 0.42^Aa^	3.60 ± 0.70^Ba^	6.40 ± 0.52^Aa^
C_70_CD	6.40 ± 0.52^Aa^	5.70 ± 0.82^ABa^	6.40 ± 0.52^Aa^	6.20 ± 0.79^Aa^	5.00 ± 0.67^Aa^	3.70 ± 0.82^Ba^	6.40 ± 0.52^Aa^
C_50_CDH	6.40 ± 0.52^Aa^	4.90 ± 0.88^Ba^	5.70 ± 0.48^Ba^	5.20 ± 0.79^Ba^	3.40 ± 0.52^Ba^	3.40 ± 0.52^Ba^	4.10 ± 0.74^Ca^
C_60_CDH	6.60 ± 0.52^Aa^	6.40 ± 0.52^Aa^	6.40 ± 0.52^Aa^	6.10 ± 0.74^ABa^	3.90 ± 0.74^Ba^	3.50 ± 0.71^Ba^	5.10 ± 0.57^Ba^
C_70_CDH	6.40 ± 0.52^Aa^	6.10 ± 0.57^Aa^	6.20 ± 0.42^ABa^	5.70 ± 0.67^ABa^	5.50 ± 0.71^Aa^	5.20 ± 0.63^Aa^	5.20 ± 0.63^Ba^
*IR drying*							
C_50_IR	5.90 ± 0.88^ABa^	5.30 ± 0.48^Aa^	6.50 ± 0.53^Aa^	6.40 ± 0.70^Aa^	4.30 ± 0.67^Ba^	3.70 ± 0.48^DEa^	5.90 ± 0.74^BCa^
C_60_IR	6.40 ± 0.52^Aa^	5.40 ± 0.52^Aa^	6.40 ± 0.52^Aa^	6.10 ± 0.88^Aa^	5.20 ± 0.42^Aa^	4.10 ± 0.74^CDa^	6.00 ± 0.47^ABCa^
C_70_IR	6.50 ± 0.53^Aa^	6.10 ± 0.88^Aa^	6.40 ± 0.52^Aa^	6.40 ± 0.52^Aa^	5.50 ± 0.53^Aa^	5.10 ± 0.74^ABa^	6.60 ± 0.52^ABa^
C_50_IRH	5.40 ± 0.52^Ba^	5.60 ± 0.52^Aa^	6.50 ± 0.53^Aa^	6.60 ± 0.52^Aa^	3.70 ± 0.67^Ba^	3.30 ± 0.48^Ea^	5.70 ± 0.82^Ca^
C_60_IRH	6.40 ± 0.52^Aa^	5.60 ± 0.70^Aa^	6.40 ± 0.52^Aa^	6.10 ± 0.88^Aa^	5.20 ± 0.42^Aa^	4.50 ± 0.53^BCa^	6.20 ± 0.42^ABCa^
C_70_IRH	6.50 ± 0.53^Aa^	6.10 ± 0.88^Aa^	6.40 ± 0.52^Aa^	6.40 ± 0.52^Aa^	5.50 ± 0.53^Aa^	5.60 ± 0.52^Aa^	6.70 ± 0.48^Aa^

* C_0_: Fresh apple slices sample, CH_0_: fresh apple slices sample infused with the *Hibiscus sabdariffa* extract, C_CD: apple slices dried by the convection method, C_CDH: apple slices infused with the *Hibiscus sabdariffa* extract dried by the convection method, C_IR: apple slices dried by the infrared (IR) method, C_IRH: apple slices infused with the *Hibiscus sabdariffa* extract dried by the IR method. Means that do not share a letter as a superscript (A–E) are significantly different at *p* < 0.05 based on the Tukey test.

The acceptability index (data not shown) showed higher values than 70%, considered as a minimum threshold for a quality characteristic, the lowest values being recorded for crispness. Higher values were obtained for the samples dried at 70°C, regardless of the method. However, when IR was applied at 70°C, the acceptability index varied from 87% for color to 96% for overall acceptability. Considering the overall acceptability, the infused slices obtained by CD showed lower values (87%) when compared with the corresponding IR slices (96%).

### 3.7 Antioxidant Activity of Apple Snacks

The DDPH antioxidant activity values of the apple snacks are given in Table S3. No significant differences (*p* < 0.05) were found between fresh samples, considered as control when compared with dried samples. Moreover, the contribution of infusion with hibiscus extract polyphenols may be observed when comparing the antioxidant activity of the control (2.21 ± 0.04 mMol/g DW) and the control samples infused with hibiscus (5.98 ± 0.06 mMol/g DW). When drying, due to the water removal and concentration, the antioxidant activity of samples decreased for the infused samples, from 5.98 ± 0.06 mMol/g DW to 2.60 ± 0.02 mMol/g DW for the infused samples dried by CD at 60°C. When comparing the values within the drying methods, it can be observed that the antioxidant values slightly decrease with the increasing temperature in all samples. These trends may be explained by the faster degradation profile of anthocyanins from sliced surfaces with temperature increase.

### 3.8 Cromatographic Profile of the Apple Snacks Dried at 70°C

The polyphenol profiles of the samples are given in Table 5. As it can be seen, the snacks dried by the two methods showed a series of common compounds, at different concentrations. These compounds are cafestol, gallic acid, catechin, protocatechuic acid, chlorogenic acid, epicatechin, ferulic acid, rutin, quercetin 3‐diglucoside, naringin, quercetin 3‐glucoside, hesperidin, quercetin, and quercetin dihydrate, and luteolin. The infused samples are differentiated by the presence of some specific compounds from the *Hibiscus* extract, such as syringic acid, epicatechin gallate, kuromanin, sinapic acid, peonidin 3‐*O*‐glucosides and peonidin 3‐*O*‐rutinoside, and ellagic acid. Regardless of the method and samples, cafestol was the main compound, with the lowest concentration in control samples dried by CD (104297.62 ± 125.03 μg/g DW) and the highest in infused samples dried by IR at 70 (127814.24 ± 111.56 μg/g DW). Catechin, epicatechin, and protocatechuic acid followed in concentration. For all the samples, significant differences (*p* < 0.05) were found between CD and IR. It is clear from the data presented that the infusion in hibiscus extract caused an increase in the polyphenolic concentrations. The differences between the two methods, with the mention of a higher concentration of compounds in the IR samples, are probably due to the reduction in drying time simultaneously with a larger amount of evaporated water. However, the higher concentration of bioactive compounds in infused samples did not interfere significantly with the antioxidant activity values.

**TABLE 5:** The bioactive profiles of the apple snacks.

Bioactive (μg/g DW)	C_70_CD	C_70_CDH	C_70_IR	C_70_IRH
Cafestol	104297.62 ± 125.03^D^	109029.33 ± 101.11^B^	107155.88 ± 99.23^C^	127814.24 ± 111.56^A^
Gallic acid	98.54 ± 2.98^D^	205.46 ± 7.62^B^	108.77 ± 3.04^C^	291.94 ± 11.12^A^
Catechin	1315.74 ± 11.24^D^	5838.95 ± 10.01^B^	2125.93 ± 18.72^C^	8629.44 ± 102.23^A^
Protocatechuic acid	323.02 ± 8.07^B^	348.59 ± 6.05^A^	324.35 ± 4.35^B^	350.69 ± 7.06^A^
Chlorogenic acid	64.59 ± 1.11^B^	67.22 ± 1.22^AB^	65.32 ± 3.08^B^	70.04 ± 1.04^A^
Epicatechin	645.63 ± 7.64^B^	729.31 ± 9.07^A^	685.94 ± 11.98^B^	758.67 ± 11.75^A^
Syringic acid	Nd	7.77 ± 1.07^A^	Nd	8.45 ± 0.95^A^
Epicatechin gallate	Nd	26.04 ± 0.99^A^	Nd	20.81 ± 1.06^B^
Ferulic acid	4.70 ± 0.99^A^	5.06 ± 0.89^A^	5.03 ± 0.58^A^	4.93 ± 0.94^A^
Rutin	5.71 ± 1.01^C^	6.88 ± 1.03^B^	6.08 ± 0.76^C^	7.21 ± 0.99^A^
Kuromanin	Nd	11.87 ± 1.12^B^	Nd	13.88 ± 1.11^A^
Sinapic acid	Nd	1.44 ± 0.45^B^	Nd	2.97 ± 0.34^A^
Quercetin 3‐diglucoside	20.92 ± 2.03^D^	29.96 ± 2.54^B^	25.31 ± 6.77^C^	32.90 ± 1.06^A^
Naringin	21.36 ± 2.01^C^	43.19 ± 3.21^B^	23.70 ± 3.12^C^	63.39 ± 2.38^A^
Quercetin 3‐glucoside	18.50 ± 2.05^C^	29.95 ± 1.87^B^	19.27 ± 1.39^C^	31.92 ± 1.38^A^
Peonidin 3‐*O*‐glucosides	Nd	47.35 ± 3.87^B^	Nd	49.44 ± 1.07^A^
Elagic acid	Nd	39.86 ± 1.03^A^	Nd	40.68 ± 2.03^A^
Hesperidin	178.92 ± 7.65^C^	236.76 ± 5.05^B^	175.11 ± 3.78^C^	262.23 ± 6.71^A^
Peonidin 3‐*O*‐rutinoside	Nd	227.25 ± 9.78^B^	Nd	236.26 ± 7.56^A^
Quercitin	36.77 ± 2.07^C^	37.95 ± 1.11^B^	36.99 ± 3.33^C^	38.04 ± 1.08^A^
Quercetin dihydrate	19.26 ± 1.22^C^	20.85 ± 1.12^B^	20.29 ± 1.11^B^	22.38 ± 0.76^A^
Luteolin	10.19 ± 0.54^C^	10.41 ± 0.56^C^	11.67 ± 0.97^B^	13.26 ± 0.78^A^

*Note:* The experiments were performed in duplicates and expressed as means±standard deviation. Superscript letters (A–D) indicate statistically significant differences between the samples with *p* < 0.05 based on the Tukey test.

Abbreviations: C_*temperature*_CD, Apple slices dried by convective air drying at 70°C; C_*temperature*_CDH, apple slices infused in the *Hibiscus sabdariffa* extract by convective air drying at 70°C; C_*temperature*_IR, apple slices dried by infrared drying at 70°C; C_*temperature*_IRH, apple slices infused in the *Hibiscus sabdariffa* extract by infrared drying at 70°C.

## 4 Conclusions

Taking into account the growing trend of snack consumption as viable options between meals, the current study focused on testing two alternative drying methods of apples with and without infusion of the *Hibiscus* extract, which is rich in polyphenols and especially anthocyanins. The purpose of infusion in polyphenolic extracts was to potentially increase the added value of the apple snacks by improving the color parameter, texture, and antioxidant capacity. CD and IR were used to obtain the apple snacks, allowing us to estimate the kinetic parameters for drying. The Page model showed the best fit for the data, whereas the kinetic parameters showed an increase in effective moisture diffusivity with temperature, and higher values were estimated for the infused sample. The activation energy value showed a higher dependence on temperature for infrared‐dried samples. The infused apple snacks showed an increase in the a* parameter, corroborated by a good sensory appreciation of color. The *BI* highly depended on the presence of the *Hibiscus* extract as potential inhibitors and temperature, as an indicator of Maillard reactions. The infused samples showed a lower firmness but increased crispness. The RR reached a lower value for infrared samples after 40 min. The SR increased with temperature for both drying methods and was higher for the infused samples obtained by CD and lower for IR. The apple snacks showed significantly higher antioxidant activity, with an increasing trend for control and a sinusoidal trend for the infused sample with increasing temperature. The samples dried at 70°C showed a common polyphenolic profile, whereas new compounds were identified in the infused sample. The main compound was cafestol, with the highest concentration in infrared samples. Our results suggest that both samples of apple snacks, with and without infusion in polyphenolic‐rich extract, may be used as adequate carriers for the incorporation of functional compounds.

## Author Contributions

**Lavinia Stan (Boldea):** data curation (equal), formal analysis (equal), methodology (equal), validation (equal), writing – original draft (equal). **Gabriel – Dănuț Mocanu:** data curation (equal), formal analysis (equal), investigation (equal), methodology (equal), validation (equal), visualization (equal), writing – original draft (equal). **Mihaela Turturică:** data curation (equal), formal analysis (equal), investigation (equal), methodology (equal), validation (equal), visualization (supporting), writing – original draft (supporting). **Doina Georgeta Andronoiu:** data curation (supporting), formal analysis (equal), investigation (equal), methodology (equal), software (equal), validation (supporting), visualization (equal), writing – original draft (supporting). **Gabriela Râpeanu:** funding acquisition (equal), resources (equal), software (equal), validation (equal), visualization (supporting), writing – original draft (supporting), writing – review and editing (equal). **Nicoleta Stănciuc:** conceptualization (lead), investigation (equal), methodology (equal), project administration (lead), resources (equal), validation (lead), visualization (equal), writing – original draft (equal), writing – review and editing (lead).

## Conflicts of Interest

All authors certify that they have participated sufficiently in the work to take public responsibility for the appropriateness of the experimental design and method, as well as the collection, analysis, and interpretation of the data. The authors have reviewed the final version of the manuscript and approved it for publication. To the best of our knowledge and belief, this manuscript has not been published in whole or in part, nor is it being considered for publication elsewhere.

## Supporting information


Data S1.

## Data Availability

The data presented in the manuscript are available on request from the corresponding author.
