# Supplementary material for: Potentially Functional Apple Snacks Infused in the Hibiscus sabdariffa Extract Obtained by Convective and Infrared Drying: Kinetics of Drying and Phytochemical Analysis
Source: Food Sci Nutr. 2025 Mar 4;13(3):e70060. doi: 10.1002/fsn3.70060 (PMC11876776; doi:10.1002/fsn3.70060)
Supplement: Supplementary file 1 — Data S1. [file FSN3-13-e70060-s001.docx]

**Tables caption**

**Table 1.** Selected mathematical models reported in the literature used for drying curves description of agricultural products

| **No.** | **Model Name** | **Mathematical Function** | **Reference** |
| --- | --- | --- | --- |
| 1. | Newton/Lewis | MR = exp(– kt) | Lewis (1921) |
| 2. | Page | MR = exp(– kt^n^) | Page (1949) |
| 4. | Henderson and Pabis | MR = a exp(– kt) | Henderson and Pabis (1962) |
| 5. | Logarithmic | MR = a exp(−kt) + c | Meisami-asl et al., (2009) |

MR – moisture ratio; n – number of constants in the model; k – drying constants (s^– 1^); a and c – coefficients and t – drying time (s)

Table 2. Results of mathematical modelling of different models used for the apple snacks

| Model name | Drying temperature  (°C) | Model parameters | | | | R^2^ | SSR |
| --- | --- | --- | --- | --- | --- | --- | --- |
|  |  | k | n | a | c |  |  |
| ***Convective drying*** | | | | | | | |
| *Control samples* | | | | | | | |
| Newton/ Lewis | 50 | 0.478 |  |  |  | 0.973 | 0.0131 |
|  | 60 | 0.704 |  |  |  | 0.962 | 0.0280 |
|  | 70 | 0.748 |  |  |  | 0.965 | 0.0592 |
| Page | 50 | 0.448 | 1.191 |  |  | 0.996 | 0.0039 |
|  | 60 | 0.641 | 1.279 |  |  | 0.995 | 0.0037 |
|  | 70 | 0.626 | 1.522 |  |  | 0.991 | 0.0051 |
| Henderson and Pabis | 50 | 0.488 |  | 1.129 |  | 0.988 | 0.0112 |
|  | 60 | 0.724 |  | 1.133 |  | 0.986 | 0.0247 |
|  | 70 | 0.788 |  | 1.145 |  | 0.981 | 0.0538 |
| Logarithmic | 50 | 0.444 |  | 1.163 | - 0.044 | 0.991 | 0.0069 |
|  | 60 | 0.587 |  | 1.219 | - 0.113 | 0.987 | 0.0134 |
|  | 70 | 0.596 |  | 1.478 | - 0.377 | 0.985 | 0.0246 |
| *Infused samples* | | | | | | | |
| Newton/ Lewis | 50 | 0.642 |  |  |  | 0.968 | 0.0515 |
|  | 60 | 0.770 |  |  |  | 0.965 | 0.0536 |
|  | 70 | 0.899 |  |  |  | 0.963 | 0.0644 |
| Page | 50 | 0.618 | 1.252 |  |  | 0.994 | 0.0213 |
|  | 60 | 0.742 | 1.264 |  |  | 0.996 | 0.0164 |
|  | 70 | 0.865 | 1.341 |  |  | 0.995 | 0.0199 |
| Henderson and Pabis | 50 | 0.564 |  | 1.074 |  | 0.986 | 0.0327 |
|  | 60 | 0.677 |  | 1.083 |  | 0.986 | 0.0340 |
|  | 70 | 0.789 |  | 1.105 |  | 0.979 | 0.0411 |
| Logarithmic | 50 | 0.546 |  | 1.084 | - 0.075 | 0.987 | 0.0327 |
|  | 60 | 0.655 |  | 1.095 | - 0.144 | 0.987 | 0.0351 |
|  | 70 | 0.764 |  | 1.126 | - 0.408 | 0.981 | 0.0513 |
| ***IR drying*** | | | | | | | |
| *Control samples* | | | | | | | |
| Newton/ Lewis | 50 | 0.0127 |  |  |  | 0.972 | 0.174 |
|  | 60 | 0.0131 |  |  |  | 0.974 | 0.189 |
|  | 70 | 0.0194 |  |  |  | 0.985 | 0.136 |
| Page | 50 | 0.0351 | 0.672 |  |  | 0.994 | 0.112 |
|  | 60 | 0.0357 | 0.701 |  |  | 0.993 | 0.113 |
|  | 70 | 0.0459 | 0.719 |  |  | 0.996 | 0.149 |
| Henderson and Pabis | 50 | 0.0125 |  | 0.907 |  | 0.975 | 0.242 |
|  | 60 | 0.0197 |  | 0.929 |  | 0.961 | 0.215 |
|  | 70 | 0.0135 |  | 0.921 |  | 0.956 | 0.286 |
| Logarithmic | 50 | 1.056 |  | 1.724 | - 0.323 | 0.957 | 0.119 |
|  | 60 | 1.042 |  | 2.176 | - 0.444 | 0.973 | 0.113 |
|  | 70 | 1.049 |  | 1.803 | - 0.343 | 0.954 | 0.136 |
| *Infused samples* | | | | | | | |
| Newton/ Lewis | 50 | 0.02 |  |  |  | 0.964 | 0.119 |
|  | 60 | 0.0286 |  |  |  | 0.973 | 0.104 |
|  | 70 | 0.0289 |  |  |  | 0.973 | 0.104 |
| Page | 50 | 0.0466 | 0.713 |  |  | 0.990 | 0.114 |
|  | 60 | 0.0546 | 0.782 |  |  | 0.992 | 0.134 |
|  | 70 | 0.0552 | 0.760 |  |  | 0.992 | 0.135 |
| Henderson and Pabis | 50 | 0.0196 |  | 0.872 |  | 0.964 | 0.233 |
|  | 60 | 0.0289 |  | 0.942 |  | 0.972 | 0.187 |
|  | 70 | 0.0292 |  | 0.953 |  | 0.973 | 0.189 |
| Logarithmic | 50 | 0.875 |  | 1.600 | - 0.331 | 0.963 | 0.116 |
|  | 60 | 0.877 |  | 1.618 | - 0.361 | 0.975 | 0.104 |
|  | 70 | 0.887 |  | 1.636 | - 0.365 | 0.976 | 0.107 |

R^2^ = coefficient of determination; SSR = sum of the residual squares; k, n, a and c = model parameters.

**Table 3**. The antioxidant activity of the apple snacks

| **Sample code*** | **Antioxidant activity,**  **mMol/g DW** |  |
| --- | --- | --- |
| ***Fresh apple slices*** | | |
| C_0_ | 2.21 ± 0.04^A^ |  |
| CH_0_ | 5.98 ± 0.06^A^ |  |
| ***Convective drying*** | | |
| C_50_CD | 2.87 ± 0.02^Aa^ |  |
| C_60_CD | 2.79 ± 0.01^Aa^ |  |
| C_70_CD | 2.79 ± 0.01^Aa^ |  |
| C_50_CDH | 3.19 ± 0.03^Aa^ |  |
| C_60_CDH | 2.60 ± 0.02^Aa^ |  |
| C_70_CDH | 2.66 ± 0.02^Aa^ |  |
| ***IR drying*** | | |
| C_50_IR | 2.87 ± 0.01^Aa^ |  |
| C_60_IR | 2.81 ± 0.02^Aa^ |  |
| C_70_IR | 2.77 ± 0.03^Aa^ |  |
| C_50_IRH | 2.91 ± 0.01^Aa^ |  |
| C_60_IRH | 2.71 ± 0.05^Aa^ |  |
| C_70_IRH | 2.68 ± 0.02^Aa^ |  |

C_*temperature*_CD – apple slices dried by convective air drying at 70°: C_*temperature*_CDH – apple slices infused in *Hibiscus sabdariffa* extract by convective air drying at 70°; C_*temperature*_IR – apple slices dried by infrared drying at 70°: C_*temperature*_IRH – apple slices infused in *Hibiscus sabdariffa* extract by infrared drying at 70°. The experiments were performed in duplicates and expressed as means±standard deviation. Within a set of experiments means at different drying treatments, on the same column that share the same superscript letter are no significantly different at p < 0.05; Within a set of experiments means at different drying temperature, on the same column that share the same lower script letter are no significantly different at p < 0.05 based on Tukey test.

**Figures caption**


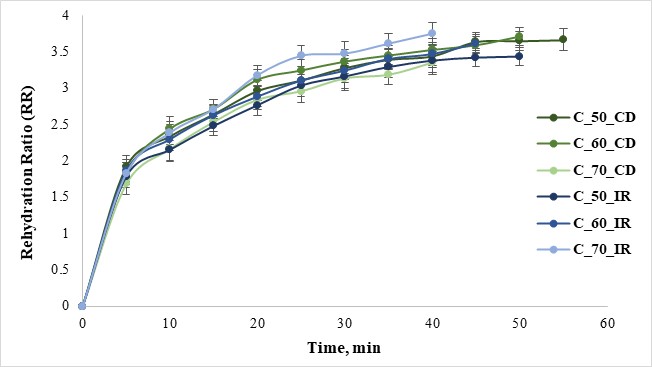

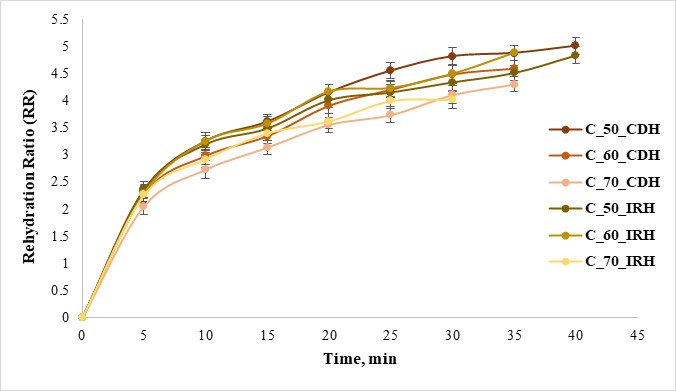


a) b)

**Figure 1.** Rehydration ratio of dried apple slices (a – apple slices convective and infrared dried,

b – apple slices infused with *Hibiscus sabdariffa* extract convective and infrared dried)


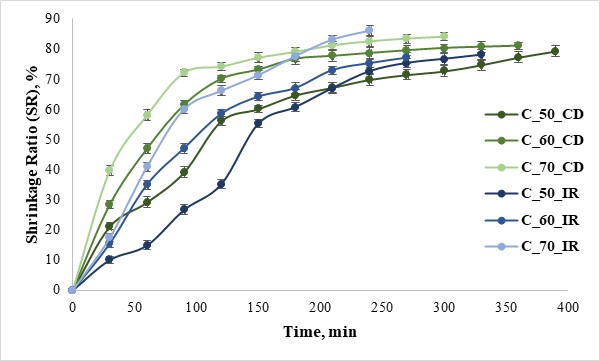

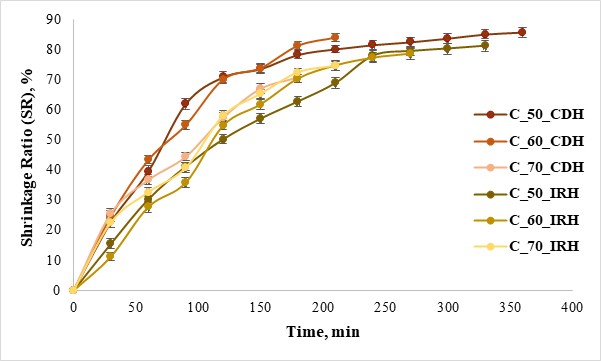


a) b)

**Figure 2.** Shrinkage ratio of dried apple slices (a – apple slices convective and infrared dried,

b – apple slices infused with *Hibiscus sabdariffa* extract convective and infrared dried)
